# C-854-08. Reducing Nursing Turnover in a Burn Trauma Stepdown Unit: A Multifaceted Leadership and Empowerment Initiative

**DOI:** 10.1093/jbcr/irag033.084

**Published:** 2026-04-06

**Authors:** Laura E Carpenter, Brooke Bozoian, Lauren A Caballero, Sherrina Richards

**Affiliations:** Warden Burn Center at Orlando Health - Orlando Regional Medical Center, Orlando, FL; Orlando Regional Medical Center, Orlando, FL; Warden Burn Center at Orlando Health - Orlando Regional Medical Center, Orlando, FL; Warden Burn Center at Orlando Health - Orlando Regional Medical Center, Orlando, FL

## Abstract

**Introduction:**

Burn units experience significantly higher nursing turnover rates compared to general hospital departments, with rates reaching up to 38% in Burn Med-Surg and 31% in Burn ICU. High turnover compromises continuity of care increases costs and contributes to staff burnout. In conjunction, low morale can lead to emotional exhaustion, especially when remaining staff must compensate for vacancies. In 2023, the Burn Trauma Stepdown Unit (BTSU) initiated a comprehensive strategy to address turnover and improve nurse retention.

**Methods:**

A multi-pronged approach was implemented, including:

- Nursing Empowerment: Autonomy and shared governance were promoted through staff-led decision-making within the Unit Nurse Practice Council, giving nurses a strong voice in shaping clinical practice and unit operations.

- Healthy Work Environment: Psychological safety and team cohesion were enhanced through intentional relationship-building, including regular check-ins and at the elbow support.

- Leadership Alignment: Leaders were strategically placed in roles that matched their strengths, enabling transformational leadership and increasing staff perception of leadership support and accessibility.

- Burn Mentor Program: Reinstated in June FY 2024, this structured mentorship initiative provided a safe space for learning, growth, and peer support for new and transitioning nurses, reinforcing clinical confidence and professional development.

- Meaningful Recognition: A culture of appreciation was embedded through celebratory programs such as The Big Dill, This is Fine Award, Employee of the Month, and Positive Potato. These initiatives honored individual contributions, teamwork, and moments of excellence, boosting morale and reinforcing positive behaviors.

**Results:**

Turnover data were tracked monthly using a rolling 24 month metric. The unit’s turnover rate decreased from 35.77% in FY 23 October to 11.34% in FY 25 August, surpassing the organizational target of 17%. The interventions led to a 68% reduction in turnover under 2 years. Research supports that mentorship programs can reduce turnover by 2–15%, and transformational leadership significantly improves retention by enhancing job satisfaction and work-life balance.

**Conclusions:**

A strategic focus on empowerment, mentorship, and leadership alignment can dramatically reduce nursing turnover in burn units. These findings support scalable models for retention in high-stress clinical environments.

**Applicability of Research to Practice:**

Burn centers should consider implementing structured mentorship and leadership development programs to improve nurse retention, reduce burnout, and enhance patient care outcomes.

**Funding for the Study:**

N/A.

